# Survival estimates of childhood malignancies treated at the Mexican telethon pediatric oncology hospital

**DOI:** 10.1002/cnr2.1702

**Published:** 2022-08-30

**Authors:** Joel Monárrez‐Espino, Lourdes Romero‐Rodriguez, Gabriela Escamilla‐Asiain, Andrea Ellis‐Irigoyen, María del Pilar Cubría‐Juárez, Douglas Sematimba, Carlos Rodríguez‐Galindo, Lourdes Vega‐Vega

**Affiliations:** ^1^ Department of Health Research, Christus Muguerza del Parque Hospital University of Monterrey Chihuahua Mexico; ^2^ PhD Program in Molecular Medicine, Human Medicine and Health Sciences Academic Unit Zacatecas Autonomous University Zacatecas Mexico; ^3^ Telethon Pediatric Oncology Hospital Querétaro Mexico; ^4^ Karolinska University Hospital Stockholm Sweden; ^5^ Department of Global Pediatric Medicine St. Jude Children's Research Hospital Memphis Tennessee USA

**Keywords:** epidemiology, hematology, oncology, pediatric cancer, relapse, survival

## Abstract

**Background:**

Pediatric cancer incidence in Mexico is ~160/million/year with leukemias making 49.8% of the cases. While survival rates have been reported in various Mexican studies, no data is available from the Telethon Pediatric Oncology Hospital‐HITO, a nonprofit private institution specialized exclusively in comprehensive pediatric oncology care in the country that closely follows high‐income countries' advanced standards of cancer care.

**Aim:**

To determine overall survival (OS) and relapse‐free survival (RFS) in patients treated at HITO between December 2013 and February 2018.

**Methods and results:**

Secondary analysis of data extracted from medical records. It included 286 children aged 0–17 years diagnosed with various cancers grouped into three categories based on location: (1) Acute lymphoblastic leukemia (ALL), (2) tumors within the central nervous system (TWCNS), and (3) tumors outside the CNS (TOCNS). OS and RFS rates for patients who completed 1 (*n* = 230) and 3 (*n* = 132) years of follow‐up after admission were computed by sex, age, and cancer location, and separately for a subsample (1‐year = 191, 3‐years = 110) who fulfilled the HITO criteria (no prior treatment, underwent surgery/chemotherapy when indicated, and initiated therapy). TOCNS accounted for 45.1%, but ALL was the most frequent single diagnosis with 28%. Three‐year OS for patients with ALL, TWCNS, and TOCNS who fulfilled the HITO criteria were 91.9%, 86.7%, and 79.3%, respectively; for 3‐year RFS these were 89.2%, 60%, and 72.4%*.* Boys showed slightly higher OS and RFS, but no major differences or trends were seen by age group.

**Conclusion:**

This study sets a relevant reference in terms of survival and relapse for children with cancer in Mexico treated at a private oncology center that uses a comprehensive and integrated therapeutic model.

## 1 INTRODUCTION

Pediatric cancer is a major challenge in low‐ and middle‐income countries (LMIC), where 80% of the cases occur,^1^ and it is becoming an emergent global health concern.^2^, ^3^

At present, cancer is the most common cause of death in children aged 5–14 years in Mexico.^4^, ^5^ Data from the National Council for the Prevention and Treatment of Childhood Cancer, a network comprising 55 public certified hospitals nationwide, revealed an incidence of pediatric cancer (0–17 years) of 156.9 per million inhabitants in 2012.^6^ Leukemia accounted for 49.8% of all pediatric cancers followed by lymphomas (9.9%), and central nervous system (CNS) tumors (9.4%) during the 2007–2012 period,^4^, ^6^ with an incidence increasing over time, particularly that of acute lymphoblastic leukemia (ALL).^7^

Several initiatives have been implemented in Mexico to establish a pediatric cancer control agenda, including the involvement of the “Seguro Popular,” the government‐funded public medical insurance program. However, efforts have faced various challenges associated with insufficient funding, ranging from limited infrastructure to poorly trained staff.^8^

Child survival from cancer in Mexico has improved considerably due to better public health interventions; yet, survival is still half of the rates observed in high‐income countries (HIC).^5^, ^7^, ^9^, ^10^, ^11^, ^12^

While survival from pediatric cancer has been linked to various factors, including genetic susceptibility, age at diagnosis, type of hospital, nutritional status, and stage at diagnosis and treatment,^12^, ^13^ in resource‐limited settings substantial improvement in survival mostly relates to adherence to integrated pediatric cancer management protocols.^14^

In this context, the aim of this study was to determine the overall survival (OS) and relapse‐free survival (RFS) rates for patients admitted to the Telethon Pediatric Oncology Hospital (HITO), a specialized nonprofit private institution that closely follows advanced standards of cancer care followed in HIC.

Written permission to use, analyze, and publish the data was given by the HITO Ethics and Research Committee (ID: CEI‐2018‐005). Informed consent was not obtained, as this was a secondary data analysis. However, complete anonymity was maintained throughout the analysis and reporting process.

## 2 METHODOLOGY

### 2.1 Study design

This was a retrospective secondary survival analysis of data extracted from medical records of pediatric patients treated for cancer at HITO. The study period lasted 50 months, starting from the hospital inauguration in December 2013 until February 2018. Standard diagnostic methods were followed as per disease condition and protocol recommendations, including imaging (radiography, ultrasound, bone scintigraphy, and CT‐PET‐MRI scans), pathology (immunohistochemistry panel, molecular pathology, cytospin CSF, FISH testing, and cytogenetics), and hematopathology (microscopy, high‐quality flow cytometry, and minimal residual disease testing) procedures.

### 2.2 Study setting

This study was carried out at a hospital located in the city of Querétaro, 200 km north of Mexico City. From the 74 pediatric cancer units in the country, this is the only hospital in Mexico exclusively focused on the treatment of pediatric cancer.^15^ It is run and owned by the Mexican Telethon Foundation, which started providing pediatric rehabilitation services in 1997, and currently runs the largest rehabilitation system for children in the world.

When HITO initiated activities, it had a medical and paramedical staff of 260; by the end of the study period, it had 350. Pediatric subspecialists in oncology, neurosurgery, oncologic surgery, transplant medicine, critical and emergency medicine, anesthesiology, radiotherapy, and radiooncology are the core medical staff, but nephrologists, endocrinologists, hematologists, pathologists, urologists, and orthopedic specialists are also affiliated.

HITO's infrastructure and services include clinical, pathological, cytogenic/molecular biology, and hematological diagnostic labs along with diagnostic/follow‐up imaging. The hospital has 26 cubicles for ambulatory chemotherapy, an Eleckta® device for radiotherapy, a hematopoietic stem cell transplantation unit, and two fully equipped operating rooms. There is a 24/7 TRIAGE/shock area, intensive care unit (ICU), and blood bank. HITO also has an adjacent 74‐room building (Casa Teletón) that provides housing and food to caretakers during the critical stages of treatment.

The hospital has an inpatient capacity of 26 beds (four for ICU) that served a yearly average of 61 new patients in active therapy between 2014 and 2017 (2014 = 58, 2015 = 78, 2016 = 50, and 2017 = 58). Mean hospital bed occupancy rate within the last 5 years was 52%. Most patients (42%) are residents of Querétaro State, but patients also come from many other Mexican states, especially from neighboring Guanajuato (15%).

Care ranges from diagnostics to reintegration to daily life. Therapeutic services offered include oncological surgery, chemotherapy, radiotherapy, and bone marrow transplantation (BMT). During the period of stay at the hospital, patients and caregivers are accommodated in Casa Teletón to minimize dropouts and for early detection of complications. Caregivers receive continuous training in numerous courses and workshops regarding cancer management.

### 2.3 Study population

The study population comprised 305 children aged 0–17 years with new diagnosis of various types of cancers during the study period. However, the final sample with complete and accurate data for analysis included 286 patients (19 patients had inconsistent diagnostic and follow‐up dates). Each medical treatment given at the hospital followed advanced standardized protocols of care used in HICs that lead to the highest possible survival, such as those of the Children's Oncology Group (COG) of the United States for solid tumors,^16^, ^17^, ^18^, ^19^, ^20^, ^21^, ^22^, ^23^, ^24^, ^25^, ^26^ and the Dana Farber Cancer Institute (DFCI) for leukemia in pediatrics.^27^, ^28^, ^29^, ^30^ These are the most experienced organizations in the world in clinical development of new therapeutics for children and adolescents. In fact, with two exceptions (i.e., DFCI protocol for ALL and St. Jude's 99 protocol for medulloblastoma), all other conditions followed COG protocols (see Supplementary Table S1). There were no ongoing research trials during the study period. Local Mexican protocols were not used, as they have not demonstrated any increase in survival rates. For all acute leukemias, antibacterial prophylaxis with levofloxacin was routinely used to reduce the incidence of bacterial infections, avoiding mortality due to sepsis during the induction phase. For all cancers, when patients presented fever and neutropenia, the antibiotic treatment protocol (mostly fourth‐generation cephalosporins) was followed within the first hour of fever. Central venous access catheters (acute or long‐term) were used throughout chemotherapy; such devices were managed by a specialized catheter clinic. Follow up care was given every month during the first year after treatment, and three or four times per year thereafter to determine the recurrence of cancer.

### 2.4 Data extraction

Data was extracted from clinical records. Due to the relatively small samples available for individual malignancies, cancers were classified into three major groups based on its location: (I) ALL (*n* = 80); (II) tumors within the central nervous system (TWCNS) (*n* = 55) including astrocytomas 23, medulloblastomas 11, ependymomas 6, adamantinomatous craniopharyngiomas 4, intracranial germ cell tumors 4, atypical teratoid/rhabdoid tumors 2, neuronal and mixed neuronal‐glial tumors 2, non‐medulloblastoma embryonal tumors 1, choroid plexus carcinomas 1, and tumors of the pineal region 1; and (III) tumors outside the CNS (TOCNS) (*n* = 132) including osteosarcomas 25, retinoblastomas 24, non‐rhabdomyosarcoma soft‐tissue sarcomas 16, rhabdomyosarcomas 12, extracranial germ cell tumors 12, neuroblastomas 10, Langerhans cell histiocytosis 9, Ewing's sarcomas 8, Wilms tumors 8, rare tumors 4, hepatoblastomas 3, and hepatocellular carcinomas 1.

Information extracted included patients' sex, age, occurrence of relapse, and status at the time of the study (i.e., death during treatment, under treatment, post‐therapy follow‐up, and abandoned). Children who received a hematopoietic stem cell transplantation (HSCT) were not analyzed (*n* = 22).

### 2.5 Statistical analysis

Frequencies and percentages were used to depict basic characteristics of the study population. RFS was defined as the time from diagnosis to disease progression. OS was defined as the time from diagnosis to death from any cause. RFS and OS as functions of time since diagnosis, were estimated using the method of Kaplan and Meier,^31^ stratifying by sex, age group (0–4, 5–9, and 10–17 years), and cancer location in patients who completed one (*n* = 230) and three (*n* = 132) years of follow‐up after admission. Kaplan–Meier plots were used to depict these graphically.

**FIGURE 1:**
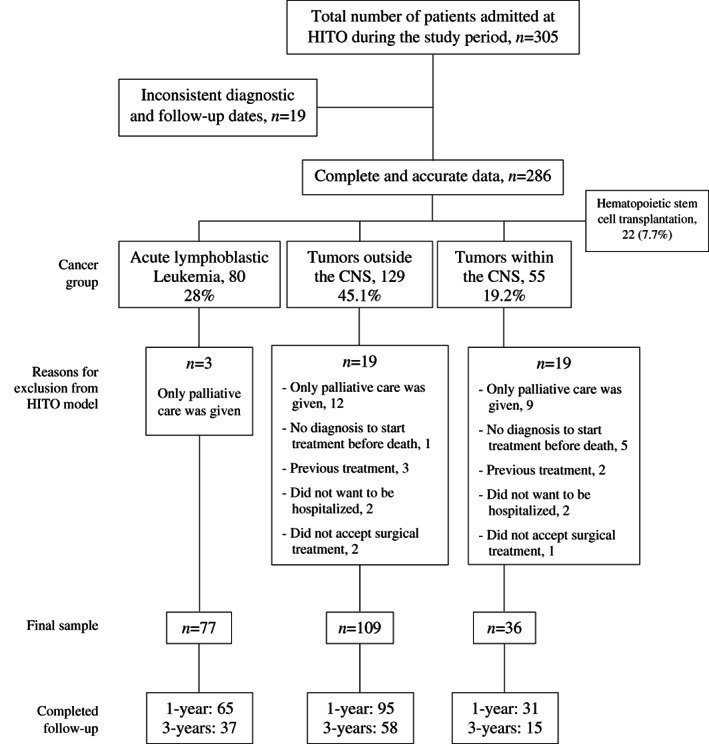
Flow‐chart showing the distribution of patients admitted to the hospital during the study period by cancer group, the number of patients with complete data excluded from the HITO model, and the final number of patients who completed the 1‐ and 3‐years follow‐up periods at the Telethon Pediatric Oncology Hospital from December 2013 to February 2018, Querétaro, Mexico

A separate analysis was conducted with patients who fulfilled the HITO criteria (i.e., no prior treatment at admission, underwent surgical/chemotherapy treatment when indicated, and initiated oncologic treatment at HITO). This was considered crucial to accurately portray the OS and RFS of patients treated with the HITO model of care, as some patients only received palliative care due to their advance stage (e.g., several patients died within few days or weeks after admission). For ALL, three patients were excluded (reason: only palliative care was given) leaving 77 available for analysis (80/77 = 96.2%). For TWCNS, 19 children had to be excluded (reasons: only palliative care was given 9, no diagnosis available to start treatment before death 5, previous treatment 2, did not want to be hospitalized 2, and did not accept surgical treatment 1), leaving 36 patients available (36/55 = 65.4%). For TOCNS, 20 patients were excluded (reasons: only palliative care was given 12, previous treatment 3, did not want to be hospitalized 2, did not accept surgical treatment 2, and no diagnosis available to start treatment before death 1) leaving 109 available (109/129 = 84.5%). Figure 1 represents a flow‐chart showing the distribution of patients admitted to the hospital during the study period by cancer group, number of patients with complete data excluded from the HITO model, and final number of patients who completed the 1‐ and 3‐years follow‐up period (Figure 1).

Patients with ALL constituted the largest group and were thus used for a sub‐analysis (*n* = 77). Cox regression analysis was used to compute crude and adjusted hazard ratios (HR) with 95% confidence intervals (CI) to identify predictors of OS and RFS in children with ALL.

Data management and statistical analyzes were carried out in SPSS v.24.

## 3 RESULTS

Table 1 presents basic characteristics of patients with cancer aged 0–17 years admitted to the hospital during the study period. Nearly 300 children were hospitalized since HITO started activities until February 2018. More number of boys were treated than girls (155 vs. 131). Children from all ages were hospitalized, but the majority were 1–4 years old (36.4%). TOCNS accounted for 45.1% of diagnoses, but ALL was the most frequent single diagnosis observed (28%). Death occurred in 22.4% of children, and relapse in 22.7% (Table 1).

**TABLE 1:** Basic characteristic of cancer patients aged 0–17 admitted to the Telethon Pediatric Oncology Hospital from December 2013 to February 2018, Querétaro, Mexico

Variable	Category	Frequency	%
Sex	Male	155	54.2
	Female	131	45.8
Age group (y)	<1	20	7.0
	1–4	104	36.4
	5–9	68	23.8
	10–17	94	32.9
Tumor location	Acute lymphoblastic leukemia	80	28.0
	Tumors within the CNS^a^	55	19.2
	Tumors outside the CNS^b^	129	45.1
	Hematopoietic stem cell transplantation	22	7.7
Relapse	Yes	65	22.7
	No	221	77.3
Current state	Death	64	22.4
	Under treatment	85	29.7
	Current	131	45.8
	Palliative care, abandoned	6	2.0
Total		286	100.0

^a^ It includes astrocytic tumors, ependymal tumors, oligodendroglial tumors, embryonal tumors, tumors of sellar region, and germ cell tumors.

^b^ It includes retinoblastomas, Wilms tumors, neuroblastomas, osteosarcomas, Ewing's sarcomas, soft‐tissue sarcomas, rhabdomyosarcomas, germ‐cell tumors, hepatic tumors, and thyroid tumors.

OS at years 1 and 3 after admission by age group, sex, and cancer location are shown in Table 2. For all cancer groups, OS at 1 and 3 years was 83.9% and 72%, respectively; boys showed consistently higher OS than girls, and children aged 10–17 years tended to have the lowest OS compared with the other age groups. For ALL, overall OS was 89.7% and 87.2% at 1 and 3 years, respectively, with OS being slightly higher in boys, and among those aged 5–9 years. OS for TWCNS were lowest compared with the other cancer groups at years 1 and 3 with 71.4%, and 59.1%, respectively, with boys having a better survival than girls. The OS rates for TOCNS were 85.8% and 67.6% at years 1 and 3, respectively, with no clear differences by sex and age group (Table 2).

**TABLE 2:** Surviving patients at years 1 (*n* = 230) and 3 (*n* = 132) after admission in children aged 0–17 years at the Telethon Pediatric Oncology Hospital from December 2013 to February 2018 by cancer location, age group and sex, Querétaro, Mexico

		Survival (%)							
Location		All ages		0–4 years		5–9 years		10–17 years	
Year	Age group	1	3	1	3	1	3	1	3
All cancers									
	Total	83.9	72.0	83.1	82.4	89.9	70.3	79.5	61.4
	Boys	87.3	76.0	84.8	85.7	92.7	73.1	84.6	66.7
	Girls	79.8	66.7	81.1	78.3	85.7	63.6	74.4	56.5
Acute lymphoblastic leukemia									
	Total	89.7	87.2	90.5	87.5	100	92.9	75.0	77.8
	Boys	97.3	91.3	100	87.5	100	90.9	90.0	100
	Girls	80.6	81.3	81.8	87.5	100	100	60.0	60.0
Tumors within the central nervous system									
	Total	71.4	59.1	58.8	75.0	77.8	50.0	78.6	50.0
	Boys	81.8	72.7	57.1	75.0	87.5	75.0	100	66.7
	Girls	63.0	45.5	60.0	75.0	70.0	25.0	57.1	33.3
Tumors outside the central nervous system									
	Total	85.8	67.6	88.9	81.5	87.5	60.0	81.8	58.6
	Boys	83.6	68.3	86.2	87.5	87.5	54.5	77.3	57.1
	Girls	89.1	66.7	93.8	72.7	87.5	75.0	86.4	60.0

Table 3 shows RFS at years 1 and 3 after diagnosis by age group, sex, and cancer location. For all cancer groups, RFS at years 1 and 3 was 85.6% and 72.5%, respectively, with boys having slightly higher rates than girls, and children aged 5–9 years also showing slightly higher rates compared with the other age groups. For ALL, RFS was 95.5% and 89.7% at 1 and 3 years, respectively, with no clear trends by sex or age group. For TWCNS, RFS was 89.7% and 61.9% at years 1 and 3, respectively, with no clear trends by sex and age group. For TOCNS, RFS was 77.8% and 66.2%, at 1 and 3 years, respectively; boys tended to have higher rates than girls, but no trend was seen by age group (Table 3).

**TABLE 3:** Patients free of relapse at years 1 (*n* = 230) and 3 (*n* = 132) after admission in children aged 0–17 years at the Telethon Pediatric Oncology Hospital from December 2013 to February 2018 by cancer location, age group, and sex, Querétaro, Mexico

		Free of relapse (%)							
Location		All ages		0–4 years		5–9 years		10–17 years	
Year	Age group	1	3	1	3	1	3	1	3
All cancers									
	Total	85.6	72.5	84.3	72.5	89.9	75.7	83.2	69.8
	Boys	85.7	74.3	84.7	75.0	92.7	76.9	79.5	70.0
	Girls	85.4	70.2	83.8	69.6	85.7	72.7	87.0	69.6
Acute lymphoblastic leukemia									
	Total	95.5	89.7	100	81.3	100	100	84.4	88.9
	Boys	91.9	91.3	100	87.5	100	100	70.0	75.0
	Girls	100	87.5	100	75.0	100	100	100	100
Tumors within the central nervous system									
	Total	89.7	61.9	94.1	62.5	83.3	62.5	92.9	60.0
	Boys	90.9	60.0	100	50.0	87.5	75.0	85.7	50.0
	Girls	88.9	63.6	90.0	75.0	80.0	50.0	100	66.7
Tumors outside the central nervous system									
	Total	77.8	66.2	73.2	70.4	83.3	60.0	79.5	65.5
	Boys	80.5	68.3	75.7	75.0	87.5	54.5	81.8	71.4
	Girls	73.9	63.3	68.8	63.6	75.0	75.0	77.3	60.0

**FIGURE 2:**
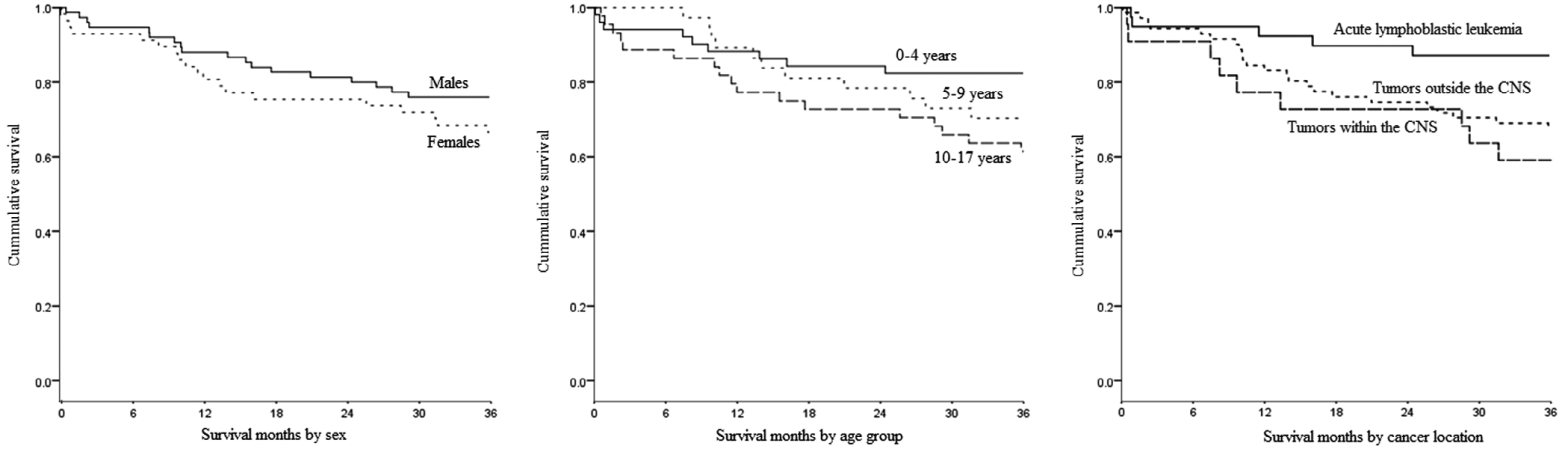
Kaplan‐Meier plots for cancer survival in months for hospitalized patients aged 0–17 years who completed 3 years of follow‐up at the Telethon Oncology Hospital from December 2013 to February 201 by sex, age group and malignancy locations, Querétaro, Mexico (*n* = 132)

Figure 2 displays Kaplan–Meier curves for OS in months by sex, age group, and cancer location. Curves depict the higher survival seen in boys, and the lower survival among those ages 0–17 years at the time of admission. For cancer location, the ALL curve reflects the notably better survival compared with the other cancer groups (Figure 2).

Table 4 presents OS and RFS for patients aged 0–17 years who fulfilled the HITO criteria at years 1 (*n* = 191) and 3 (*n* = 110) after admission. OS was higher in this subsample compared with the full sample for all cancer groups in both follow‐up years analyzed (e.g., year 1: 94.2% vs. 83.9%; year 3: 84.5% vs. 72%). Overall, ALL reached 93.8% and 91.9% survival, TWCNS 90.3% and 86.7%, and TOCNS 95.8% and 79.3% at years 1 and 3, respectively. Both 1‐ and 3‐year survival rates tended to decrease with age group. For FRS, overall rates were slightly higher for years 1 and 3 when using the HITO criteria than with the full sample (year 1: 87.9% vs. 85.9%; year 3: 76.4% vs. 72.5%). Children with ALL reached 95.3% and 89.2% free of relapse, TWCNS 87.1% and 60%, and TOCNS 83.1% and 72.4% at years 1 and 3, respectively. Neither OS nor RFS showed clear trends in rates by age group (Table 4).

**TABLE 4:** Surviving and free of relapse patients who fulfilled the HITO criteria^a^ at years 1 (*n* = 191) and 3 (*n* = 110) at the Telethon Pediatric Oncology Hospital from December 2013 to February 2018 by cancer location and age group, Querétaro, Mexico

		Percentage							
Location		All ages		0–4 years		5–9 years		10–17 years	
Year	Age group	1	3	1	3	1	3	1	3
Survival									
All cancers		94.2	84.5	95.8	91.3	94.8	81.3	91.8	78.1
Acute lymphoblastic leukemia		93.8	91.9	100	93.3	100	92.9	87.5	78.9
Tumors within the CNS		90.3	86.7	90.9	100	81.8	66.7	100	100
Tumors outside the CNS		95.8	79.3	95.2	88.0	95.0	75.0	97.0	71.4
Free of relapse									
All cancers		87.9	76.4	84.7	71.7	93.1	84.4	86.8	75.0
Acute lymphoblastic leukemia		95.3	89.2	100	80.0	100	100	83.6	85.7
Tumors within the CNS		87.1	60.0	90.9	50.0	81.8	66.7	88.9	66.7
Tumors outside the CNS		83.1	72.4	76.0	72.0	90.0	75.0	87.9	71.4

^a^ No prior treatment received, underwent surgery/chemotherapy when indicated, and initiated therapy at the hospital.

Crude and adjusted HRs with 95% CIs for surviving and free of relapse children with ALL aged 0–17 years who fulfilled the HITO criteria (*n* = 77) are shown in Table 5. Sex, age group, and risk category were not significant predictors in both crude and adjusted analyzes for either outcome. However, adjusted HR point estimates for survival were higher as the cancer risk category increased (high 3.34, very high 4.77), and among girls (1.96); similar patterns were also seen for relapse‐free HRs (Table 5).

**TABLE 5:** Crude and adjusted hazard ratios (HR) with 95% confidence intervals (CI) from Cox regression for relapse‐free and overall survival of children with acute lymphoblastic leukemia aged 0–17 years (*n* = 77) who fulfilled the HITO criteria^a^ at the Telethon Pediatric Oncology Hospital from December 2013 to February 2018, Querétaro, Mexico

Model	Category	HR (95% CI)	
		Crude	Adjusted^b^
Overall survival			
Risk category	Standard	1.00	1.00
	High	5.87 (0.65–52.6)	3.34 (0.24–44.7)
	Very high	7.57 (0.84–67.8)	4.77 (0.49–45.8)
Sex	Male	1.00	1.00
	Female	1.90 (0.51–7.11)	1.96 (0.50–7.66)
Age group in years	1–4	1.00	1.00
	5–9	0.20 (0.02–1.81)	0.31 (0.03–3.12)
	10–17	1.49 (0.37–5.98)	1.36 (0.20–9.17)
Relapse‐free			
Risk category	Standard	1.00	1.00
	High	4.19 (0.84–20.7)	2.84 (0.38–21.1)
	Very high	4.68 (0.90–24.1)	4.77 (0.85–26.6)
Sex	Male	1.00	1.00
	Female	0.97 (0.31–2.98)	0.98 (0.31–3.06)
Age group in years	1–4	1.00	1.00
	5–9	0.79 (0.19–3.18)	1.19 (0.26–5.38)
	10–17	1.99 (0.53–7.49)	1.93 (0.28–13.0)

^a^ No prior treatment, underwent surgery/chemotherapy when indicated, and initiated therapy at the hospital.

^b^ The models were adjusted for all the variables included in the table.

## 4 DISCUSSION

This study aimed at determining OS and RFS rates for patients admitted at HITO between 2013 and 2018. OS was used as an indicator of the overall care provided, and RFS as a measure of success of the initial treatment.

Of all malignancies studied, 28% corresponded to ALL, 19.2% to TWCNS, and 45.1% to TOCNS. These proportions are similar to the reported in the international literature.^32^

From all hematological malignancies admitted at HITO during the study period (*n* = 116), 80 were ALL and 36 were non‐ALL (Hodgkin lymphoma 12, non‐Hodgkin lymphoma 11, acute myeloid leukemia 10, and chronic myeloid leukemia 3). From the leukemias, the proportion of ALL was 86%, very similar to the 85.1% reported in a study with children aged <15 years diagnosed in public hospitals of Mexico City during 2006–2007.^33^ OS and RFS for all hemathological malignancies was 88.5% and 82.9% at year 3, respectively. When excluding patients not enrolled in the HITO model, the sample for each non‐ALL malignancy was further reduced. Therefore, we decided to present ALL as a single group to establish comparisons with other populations, as this is the most frequent pediatric cancer.

We pooled together TOCNS due to sample size constraints, and because this is the official categorization of the “Seguro Popular,”^34^ the governmental institution that shares with HITO the financial costs of the care provided to 64.5% of the patients admitted.

OS of HITO children treated between December 2013 and February 2018 with ALL was higher compared with that of other cancers, as previously reported.^35^, ^36^ The 50%–65% 5‐year OS for ALL reported in Mexico among children aged 0–17 years^37^ contrasts with the notably higher 91.9% observed under the HITO model at 3‐years of follow‐up. This survival can also be compared with a study from the University Hospital of Monterrey (northern Mexico) where 5‐year survival of patients aged ≤16 years treated with BFM‐inspired protocols during the 2004–2015 period for ALL ranged 55.5%–67.1%.^38^ In another study with an average follow‐up of 3.9 years, children aged <16 years treated between 2006 and 2010 at the National Medical Center of the Mexican Institute of Social Security for ALL using a DFCI protocol showed a survival of 63.9%.^39^ HITO's survival is comparable to that reported by the COG Report that contains data ranging from 1982 to 2002.^40^ In fact, the OS for pediatric patients at HITO for standard risk ALL matched the benchmark estimates achieved in high‐income settings.^41^

HITO patients also showed lower ALL relapse rates compared with other Mexican studies published earlier.^37^, ^39^, ^42^ This is likely due to the better quality of care and to the higher adherence to HIC's advanced standards of care.

Early diagnosis and treatment of ALL relate to better OS and RFS.^43^ However, patients aged <1 year and > 10 years tend to have less favorable outcomes.^44^, ^45^ During the 3‐year follow‐up period, children aged 0–4 years relapsed notably less than older children, a finding consistent with recent studies that report better survival outcomes in those with no early relapse,^46^ and among younger children.^47^

On the other hand, some differentials by sex with regards to ALL survival and relapse were observed, as it has been reported by other authors.^48^, ^49^ In this study, sex differences were relatively small not reaching statistical significance in log‐rank tests (e.g., 3‐year OS for all cancers in those aged 0–17 years: boys 76% vs. girls 66.7%; *p* > 0.05); differences seemed to relate to the small sample, especially when stratifying data. Also, crude (HR 1.90; 95% CI 0.51–7.11) and adjusted (1.96; 0.50–7.66) estimates for sex were not significant in regression analyzes. From the cultural perspective, we have no data suggesting a possible parental bias whereby caretakers might give more value and care to boys. Financially, this can be ruled out, as the full treatment given, including the parents' support in the adjacent facilities devised for that purpose, were completely free of charge.

We tried to identify predictors of survival and relapse in patients with ALL using Cox regression. Risk category is a well‐established predictor of survival with higher HR in the high‐risk and very high‐risk categories compared with the standard‐risk category.^27^, ^50^ Also, adolescents have shown higher HR compared with younger children.^48^ However, neither sex, age group, nor risk category were statistically associated in adjusted analyzes. Possible reasons for failing to replicate these findings include the small sample size that led to low precision, and the incomplete adjustment of confounders including tumor subtype and staging, and the initial response to therapy that could have biased the estimates.

Based on the HITO model, 1‐year relapse rate was 4.7%, 12.9%, and 16.9% for ALL, TWCNS, and TOCNS, respectively. All patients who relapsed after the first year were given a second‐line treatment with the intention to cure.^51^, ^52^, ^53^ Comparing these results with international studies could not be done, as we followed the Mexican official classification, and combined various diagnoses due to sample size constraints.

We decided to conduct a specific analysis with patients who fulfilled the HITO criteria. This was done to better assess the therapeutic performance of the HITO model. After exclusion, OS tended to increase, as one would have expected (e.g., all cancers, all ages, 3‐year OS: non‐HITO 72% vs. HITO 84.5%; ALL, all ages, 3‐year OS: non‐HITO 87.2% vs. HITO 91.9%). This finding points to the value of using an integrated and comprehensive care model.^54^, ^55^ The HITO model uses a family‐centered strategy that prevents treatment abandonment and promotes comprehensive health care; it is supported in five pillars: (1) diagnostic certainty to give the best treatment for each patient, (2) advanced evidence‐based medicine for a successful therapy, (3) human development at Casa Teletón to encourage personal growth through educational and occupational activities that promote treatment adherence, (4) improved quality of life for patients/caretakers to advance their physical, emotional, and spiritual well‐being aimed at avoiding treatment abandonment through services such as psychooncology, thanatology, recreational activities, schooling program, palliative care, and pain management, and (5) prevention and management of complications to avoid deaths using an infection control program, and the 24/7 functioning of the ICU and blood bank to treat and mitigate side effects and complications.

Compared with the full analyzes, the sub‐analysis showed higher OS, especially for TWCNS. This indicates that excluded patients had a considerably poorer prognosis due to an advanced cancer stage without proper diagnosis/treatment, and/or because therapy used initially was not followed. These were patients that did not accept the standardized oncologic therapy (i.e., did not consent chemotherapy and/or surgery), had been previously treated elsewhere, or died before an accurate histopathological diagnosis was established. Regardless of whether patients followed the HITO model or not, all children received the best available treatment at the time of initial assessment according to the consensus of the specialists' working group.

While treatment duration for most conditions diagnosed at HITO is at least 1 year, 1‐year survival was included because mortality due to chemotherapy complications within the first months of treatment in LMIC can reach 23% compared with 3% in HIC. Mortality has been associated with inadequate nutrition and poor hospital accessibility. After initial treatment, children from low‐income settings are sent to their homes located far away, and with poor access to hospital facilities. These hospitals are often deprived of means to handle severe complications caused by the toxicity of the intense induction chemotherapy.^56^, ^57^, ^58^, ^59^ Actually, in a Mexican study of children with ALL treated using a DFCI protocol, 7% died during induction; deaths were associated with delays in hospital care, poor general conditions, tumor charge, malnutrition, infection, and treatment toxicity.^41^ Moreover, a recent study from a major pediatric hospital in Mexico revealed the importance of improving the safety and quality of care of children with ALL; it was estimated that the incidence of adverse events (51.8/1000 patient‐days) linked to morbidity and mortality during the induction phase that can be prevented and mitigated was 10.5% and 53.6%, respectively.^60^

Early relapse has also been used to predict survival.^46^, ^51^, ^52^, ^53^ While no such correlation was observed for ALL using 18‐month relapse in this study, a moderate correlation was seen between 12‐month relapse and survival for TOCNS (*r* = 0.44) and TWCNS (*r* = 0.43).

Results from HSCT patients were not presented, as these patients belonged to two different groups: those who were initially treated using the HITO model and then transplanted, and those transferred from another institution just for the transplant. Patients from the latter group, who represented more than half of all HSCT, had incomplete data regarding the previous treatment received. Therefore, the scant number of HSCT HITO patients with complete data precluded meaningful estimates. All trasplants were allogeneic of related donors (46%), autologous (27%), and haploidentical (27%). Initial diagnoses included mostly ALL in second remission (*n* = 9), high risk neuroblastoma (*n* = 5), and chronic myelogenous leukemia (*n* = 3). The100‐day post‐transplant survival was 100%, and post‐transplant relapse was 27% during the study period.

The major strength of HITO relates to the standard set in Mexico, in terms of OS and RFS, by a specialized hospital exclusively focusing on pediatric oncology care using a comprehensive and integrated therapeutic model. This hospital is part of a nonprofit foundation that provides nondiscriminatory pediatric oncology care to children from all socioeconomic strata from any Mexican state, but mostly to the poor. Actually, 98% of patients treated at HITO are not affiliated to any public or private health institution. Another strength relates to the 2% treatment abandonment. This very low rate is explained by three main factors: to Casa Teletón that provides comprehensive assistance to children/caretakers during critical stages of treatment, to the fact that all care provided is completely free of charge, and to the work conducted by social workers that provide financial assistance to patients and caretakers to attend scheduled visits in the case of economic hardships.

Finally, some limitations ought to be mentioned: (1) the sample size for individual cancers other than ALL were too small, especially for TWCNS, to produce stable survival estimates; the 19 patients excluded initially due to incomplete or inaccurate data were the result of missing data, typing mistakes, and incoherent information, and are therefore unlikely to have biased the results observed, (2) the grouping of cancers within and outside the CNS precluded direct comparisons with other studies that do not use such categorization; it was used, as it is the official classification of the Mexican Secretariat of Health,^61^ (3) the inability to compute 5‐year survival estimates, as only a small number of patients had completed such follow‐up when permission was granted to retrieve the data for analyzes, (4) the incomplete number of variables required to run a more thorough adjusted analysis for ALL, and (5) the limited representativeness of the sample, which makes it difficult to generalize the findings to public hospitals with limited resources, and to private settings not specialized in pediatric oncology care.

In spite of increasing funding to treat pediatric cancer in Mexico, survival has not increased as expected.^62^ Poor survival in LMIC is often related to delayed, erroneous, or deficient diagnosis, lack of specialized facilities and services, treatment abandonment, lack of adequate medications, treatment toxicity, excess relapse, malnutrition, and high prevalence of comorbidities.^63^ While HITO has been able to provide advanced treatment and supportive care throughout the illness process, unfortunately many pediatric oncologic units in Mexico lack of adequate infrastructure, services, and trained staff to offer adequate care that can lead to the highest survival possible. Therefore, it is crucial that governmental and private health institutions, along with philanthropic organizations, collaborate together from the early identification of children with cancer to all subsequent therapeutic and rehabilitation stages.

We believe that the findings presented here highlight the value of implementing a successful therapeutic model in a country with relatively limited resources such as Mexico that could be of use in similar settings.

## AUTHOR CONTRIBUTIONS

**Joel Monárrez‐Espino:** Conceptualization (lead); data curation (supporting); formal analysis (lead); investigation (lead); methodology (lead); project administration (equal); supervision (equal); validation (equal); writing – original draft (lead); writing – review and editing (lead). **Lourdes Romero‐Rodríguez:** Conceptualization (supporting); data curation (lead); formal analysis (supporting); investigation (equal); methodology (equal); project administration (equal); validation (equal); writing – review and editing (equal). **Gabriela Escamilla‐Asiain:** Conceptualization (lead); data curation (equal); investigation (equal); methodology (equal); project administration (lead); supervision (lead); writing – review and editing (equal). **Andrea Ellis‐Irigoyen:** Data curation (supporting); investigation (supporting); methodology (supporting); validation (supporting); writing – review and editing (supporting). **María del Pilar Cubría‐Juárez:** Data curation (supporting); investigation (supporting); methodology (supporting); validation (supporting); writing – review and editing (supporting). **Douglas Sematimba:** Data curation (supporting); methodology (supporting); validation (supporting); writing – review and editing (supporting). **Carlos Rodríguez‐Galindo:** Data curation (supporting); methodology (supporting); validation (supporting); writing – review and editing (supporting). **Lourdes Vega‐Vega:** Conceptualization (equal); investigation (supporting); methodology (supporting); supervision (supporting); writing – review and editing (supporting).

## ETHIC STATEMENT

The following sentences were added: All procedures preformed were in accordance with national and international ethical standards.

## CONFLICT OF INTEREST

The authors declare no conflict of interest.

## Supporting information


**SUPPLEMENTARY TABLE S1** Summary of the treatment protocols used by type of pediatric malignancy at HITO, Querétaro, Mexico

## Data Availability

The data that support the findings of this study are available on request from the corresponding author. The data are not publicly available due to privacy or ethical restrictions.
