# Supplementary material for: Survival estimates of childhood malignancies treated at the Mexican telethon pediatric oncology hospital
Source: Cancer Rep (Hoboken). 2022 Aug 30;6(2):e1702. doi: 10.1002/cnr2.1702 (PMC9939997; doi:10.1002/cnr2.1702)
Supplement: Supplementary file 1 — SUPPLEMENTARY TABLE S1 Summary of the treatment protocols used by type of pediatric malignancy at HITO, Querétaro, Mexico [file CNR2-6-e1702-s001.docx]

| **SUPPLEMENTARY TABLE.** Summary of the treatment protocols used by type of pediatric malignancy at HITO, Querétaro, Mexico. | | |
| --- | --- | --- |
| Group | Malignancy | Treatment protocol |
| Malignant hemathopathy | Acute lymphoblastic leukemia | DFCI ALL consortium protocol 05-001 |
|  | Acute myelogenous leukemia | AML02: A collaborative trial for the treatment of patients with newly diagnosed acute myeloid leukemia or myelodysplasia |
|  | Chronic myelogenous leukemia | Targeted therapy with tyrosine kinase inhibitors (imatinib, dasatinib) |
|  | Non-Hodgkin lymphoma | FAB/LMB-96 regimen with or without rituximab |
|  | Hodgkin lymphoma | VAMP protocol for patients with low-risk disease, OEPA-COPDAC protocol for patients with intermediated or high-risk disease |
| TOCNS | Osteosarcoma | Cisplatin, doxorubicin, and high-dose methotrexate regimen (MAP) |
|  | Retinoblastoma | Eye-salvage treatment for small tumor with local therapy (laser therapy); enucleation for large intraocular tumors with or without adjuvant chemotherapy (vincristine, carboplatin, and etoposide). Cisplatin-based regimens, with consolidation using high-dose chemotherapy and autologous hematopoietic stem cell rescue for patients with extraocular and metastatic disease. |
|  | Rhabdomyosarcoma | COG approach (ARST0331 for low-risk embryonal/botyroid/spindle cell rhabdomyosarcoma; ARST0531 for intermediate-risk rhabdomyosarcoma, ARST0431 for high-risk rhabdomyosarcoma |
|  | Ewing's sarcoma | COG AEWS1031 protocol regimen A |
|  | Non-rhabdomyosar. soft-tissue sarcoma | COG ARST0332 protocol |
|  | Wilms tumor | COG RTS approach (regimen EE-4A, regimen DD-4A, regimen I) |
|  | Extracranial germ cell tumors | Bleomycin, etoposide, and cisplatin (BEP) standard chemotherapy regimen |
|  | Neuroblastoma | COG approach (observation or resection for low-risk, ANBL0531 for intermediate-risk, ANBL0532 for high-risk) |
|  | Hepatoblastoma | SIOPEL 3-SR protocol with six courses of cisplatin for standard-risk patients, SIOPEL-4 protocol with dose-dense cisplatin/doxorubicin chemotherapy and radical surgery for patients with high-risk hepatoblastoma |
|  | Hepatocellular carcinoma | Complete surgical resection of the primary tumor, if possible, followed by chemotherapy (Cisplatin and doxorubicin) |
|  | Langerhans cell histiocytosis | HISTSOC-LCH-III (NCT00276757) protocol |
|  | Rare tumors | No standard care of treatment; individualized therapy protocol |
| TWCNS | Astrocytoma | LGG Surgery VCR / CBP, HGG surgery/temozolomide |
|  | Ependymoma | Children’s Oncology Group trial ACNS0121 conformal radiation therapy for pediatric ependymoma, chemotherapy for incompletely resected |
|  | Choroid plexus carcinoma | CPT-SIOP-2009 |
|  | Neuronal/mixed neuronal-glial tumors | No standard care of treatment; individualized therapy protocol |
|  | Tumors of the pineal region | No standard care of treatment; individualized therapy protocol |
|  | Medulloblastoma | St Jude Protocol 99 for medulloblastoma high Risk. COG A9961 B medulloblastoma |
|  | Non-medulloblastoma embryonal tumor | Craniospinal radiotherapy followed by one adjuvant chemotherapy regimens with CPM, CDDP, VCR in children with newly-diagnosed average-risk medulloblastoma; St Jude Protocol 99 for medulloblastoma high risk |
|  | Atypical teratoid/rhabdoid tumor | ACNS0333 treatment of atypical teratoid/rhabdoid tumors (AT/RT) of the central nervous system with surgery, intensive chemotherapy, and 3-D conformal radiation |
|  | Adamantinomatous craniopharyngioma | Surgery and radiotherapy |
|  | Intracranial germ cell tumors | SIOP CNS GCT 96 chemotherapy followed by focal primary site irradiation for patients with localized disease, metastatic patients get 24 Gy craniospinal radiotherapy with 16 Gy boost to the primary site and metastases with additional chemotherapy |
